# LPS-induced Pellino3 degradation is mediated by p62-dependent autophagy

**DOI:** 10.1186/cc11737

**Published:** 2012-11-14

**Authors:** A Heeg, L Kuchler, LK Eifler, T Knape, H Heide, B Brüne, A von Knethen

**Affiliations:** 1 Institute of Biochemistry I - Pathobiochemistry, Goethe-University Frankfurt, Germany; 2 Fraunhofer IME Project Group - Translational Medicine and Pharmacology, Frankfurt, Germany; 3 Molecular Bioenergetics - Centre of Biological Chemistry, Goethe-University Frankfurt, Germany

## Background

In macrophages Toll-like receptor 4 (TLR4) is activated in response to lipopolysaccharide (LPS) and induces proinflammatory cytokine expression [1]. Therefore, mechanisms terminating proinflammatory gene expression are important. Autophagy plays a central role in controlling innate immune responses by lysosomal degradation of signaling proteins, thus contributing to the resolution of inflammation [2]. Autophagic proteins like p62 directly interact with molecules involved in the TLR4-signaling pathway, but a correlation with the IRAK E3 ligase and scaffold protein Pellino3 remains obscure [3,4]. Hence, we are interested in elucidating the function of Pellino3 to prove our hypothesis that it is a key regulator in the TLR4-signaling cascade [5].

## Methods

We used the cecal ligation and puncture (CLP) mouse model causing polymicrobial sepsis to analyze Pellino3 protein and mRNA expression. Furthermore, we induced endotoxemia in RAW264.7 mouse macrophages by LPS treatment to verify *in vivo *experiments. Lentiviral Pellino3 knockdown in RAW264.7 macrophages was used for cytokine measurements at mRNA level. To analyze potential Pellino3 binding partners in TLR4-signaling by mass spectrometry (MS), we overexpressed FLAG-tagged Pellino3 in RAW264.7 macrophages, treated cells for 3, 6 and 24 hours with LPS and immunoprecipitated Pellino3 via its FLAG-tag. To consider Pellino3 degradation as a result of p62-mediated autophagy, we transiently knocked down p62 by siRNA in RAW264.7 macrophages and also pharmacologically blocked LPS-induced autophagy by Bafilomycin A1.

## Results

We demonstrated Pellino3 protein degradation in primary CD11b^+ ^splenocytes after 24 hours following CLP operation and confirmed this in RAW264.7 macrophages after 24-hour LPS stimulation. Knockdown of Pellino3 attenuates proinflammatory cytokines, for example IL-6 mRNA, after 6 hours of LPS. Furthermore, we found by MS and verifying immunoprecipitation experiments that p62 is a Pellino3 binding partner, thus targeting Pellino3 for degradation. In line, both p62 knockdown and Bafilomycin A1 treatment prevent Pellino3 degradation, supporting an autophagic mechanism.

## Conclusion

Our observations highlight a regulatory role of Pellino3 on TLR4 signaling. Thus, antagonism of Pellino3 in the hyperinflammatory phase of sepsis may counteract the cytokine storm. Furthermore, stabilization of Pellino3 by inhibition of autophagy in the hypoinflammatory phase of sepsis may improve immunity. In consideration of these two conflictive sepsis phases, modulation of Pellino3 may provide a new strategy for the development of a therapy approach in sepsis.

## References

[B1] Zhu J Mohan C Toll-like receptor signaling pathways - therapeutic opportunities Mediators Inflamm 2010 2010 781235 20981241 10.1155/2010/781235 PMC2963142

[B2] Xu Y Liu XD Gong X Eissa NT Signaling pathway of autophagy associated with innate immunity Autophagy 2008 4 110 112 18059159 10.4161/auto.5225

[B3] Levine B Mizushima N Virgin HW Autophagy in immunity and inflammation Nature 2011 469 323 335 10.1038/nature09782 21248839 PMC3131688

[B4] Schauvliege R Janssens S Beyaert R Pellino proteins are more than scaffold proteins in TLR/IL-1R signalling: a role as novel RING E3-ubiquitin-ligases FEBS Lett 2006 580 4697 4702 10.1016/j.febslet.2006.07.046 16884718

[B5] Hennessy EJ Parker AE O'Neill LA Targeting Toll-like receptors: emerging therapeutics? Nat Rev Drug Discov 2010 9 293 307 10.1038/nrd3203 20380038

